# Clinicopathological Significances and Prognostic Value of *PPFIA4* in Colorectal Cancer

**DOI:** 10.7150/jca.78634

**Published:** 2023-01-01

**Authors:** Fangmei Fu, Rui Niu, Minying Zheng, Xiaohui Yang, Linlin Fan, Wenzheng Fu, Shiwu Zhang

**Affiliations:** 1 Department of Pathology, Tianjin Union Medical Center, Nankai University, Tianjin, 300121, P.R. China.; 2 Graduate School, Tianjin University of Traditional Chinese Medicine, Tianjin, 301617, P.R. China.; 3 Nankai University School of Medicine, Nankai University, Tianjin, 300071, P.R. China.; 4 Department of Colorectal Surgery, Tianjin Union Medical Center, Tianjin, P.R. China.

**Keywords:** Colorectal cancer, PPFIA4, MiR-485-5p, Bioinformatics Analysis, Prognosis

## Abstract

**Purpose:** The *PPFIA* gene family (*PPFIA1, PPFIA2, PPFIA3,* and *PPFIA4*) is associated with multiple human diseases, particularly malignant tumors. However, the expression and prognostic value of the *PPFIA* family in human colorectal cancers (CRCs) have not been reported.

**Materials and methods:** In this study, several databases, including Oncomine, UALCAN, and the cancer cell line encyclopedia, were used to compare differences in *PPFIA1, PPFIA2, PPFIA3*, and *PPFIA4* expression between normal colon samples and CRCs. The expression levels of these four proteins were used to evaluate the survival of patients with CRC, as determined by the Cancer Genome Atlas Program (TCGA) portal and gene expression profiling interactive analysis (GEPIA) databases. Western blotting and reverse transcription-polymerase chain reaction were performed to detect protein and mRNA levels of *PPFIA1, PPFIA3,* and *PPFIA4,* respectively. Immunohistochemical (IHC) staining was used to detect the correlation between *PPFIA4* expression and the degree of CRC malignancy. Furthermore, potential miRNAs targeting *PPFIA4* in CRCs were studied and confirmed.

**Results:** Bioinformatic analysis showed that the mRNA levels of *PPFIA1, PPFIA3*, and *PPFIA4* were higher in CRC tissue samples than in normal colon tissue. Both mRNA and protein expression of *PPFIA1, PPFIA3*, and *PPFIA4* were increased in the CRC cell lines LoVo and Hct116 compared with the normal colon epithelial cell line. Only *PPFIA4* was associated with the prognosis of patients with CRC, which was confirmed by TCGA portal and GEPIA. IHC staining confirmed that the expression of *PPFIA4* was higher in CRC tissues than in normal colon tissues and also increased in poorly differentiated CRC tissues and lymph node metastatic foci in comparison with well-differentiated CRC tissues and moderately differentiated CRC tissues. Functional annotation enrichment analysis indicated that the top 100 genes co-expressed with *PPFIA4* were enriched in the G-protein coupled peptide receptor activity, leukotrience B4 receptor activity, and peroxisome proliferator-activated receptors and hypoxia-inducible factor-1 signaling pathways. In addition, miR-485-5p negatively regulates the expression of *PPFIA4*.

**Conclusion:** *PPFIA4* expression is associated with the development of CRCs and may be a novel potential prognostic marker for human CRCs.

## Introduction

Colorectal cancer (CRC) is the second most common cause of death and the third most common cause of malignant tumors 1, 2. The analysis of differentially expressed genes between CRC and normal colon tissues may provide new targets for cancer research. Substantial biological information was identified in the post-genome era by biological information scientists, who also established a good deal of software and databases for systematic management and sufficient sequence data to study the molecular mechanisms of tumor initiation and progression. Additionally, the development of bioinformatics contributes to the search for appropriate biomarkers for tumor prognosis.

The *PPFIA* genes, which include *PPFIA1, PPFIA2, PPFIA3* and *PPFIA4*, were discovered by encoding liprin-α family member proteins, which were discovered by interacting with the leukocyte common antigen-related gene (LAR) family 3, 4. These four genes play important roles in the initiation and progression of tumors. Liprin-α1, encoded by *PPFIA1*, has the highest degree of alternative splicing in this family and is overexpressed in several epithelial cancers, including head and neck, breast, and ovarian cancers 5-8. PPFIA2 expression is increased in the urinary sediments of patients with prostate cancer 9. PPFIA3 is a significant marker of gastric cancer and has independent prognostic value in patients with pancreatic cancer 10-12. Liprin-α4, encoded by PPFIA4, is located on chromosome 1q32. Overexpression of this protein promotes pancreatic cancer cells' proliferative and invasive abilities, whereas inhibition of this protein's expression improves the therapeutic effect in small cell lung cancer 13-15. Liprin-α4 may be a novel therapeutic target for malignant tumors.

In this study, the expression of different *PPFIA* genes in normal colon samples and CRC tissues were analyzed using several databases, including Oncomine, UALCAN, and the cancer cell line encyclopedia (CCLE). Our results showed that *PPFIA1, PPFIA3*, and *PPFIA4* expression were higher in colon cancer cell lines (LoVo and Hct116) than in the normal colon epithelial cell line. Among the four genes, *PPFIA4* was associated with the prognosis of patients with CRC based on TCGAportal and gene expression profiling interactive analysis (GEPIA). Functional annotation enrichment analysis indicated that *PPFIA4* is involved in the peroxisome proliferator-activated receptors (PPAR) and hypoxia-inducible factor-1 (HIF-1) signaling pathways, and miR-485-5p negatively regulates the expression of *PPFIA4*.

## Materials and methods

### 1.*PPFIA*s expression levels analysis

UALCAN (http://ualcan.path.uab.edu) and Oncomine (https://www.oncomine.org/) databases were used to investigate the mRNA expression of PPFIA in normal colon and CRC samples. The criteria for the inclusion of patients downloaded from the database included: (1) patients did not receive chemotherapy and/or radiotherapy before radical operation; (2) patients did not accompany any other tumors; (3) patients' age was at least 18 years. The criteria for exclusion included: (1) The clinicopathologic data of patients was incomplete; (2) patients' age was less than 18 years. CCLE datasets were used to verify the mRNA expression levels of *PPFIA* genes in different cancer cell lines (https://sites.broadinstitute.org/ccle/). The Human Protein Atlas database was used to detect protein expression of *PPFIA4* in normal colon and CRC tissues.

### 2. Cancer cell lines and culture

The normal human colon cell line NCM460 and colon cancer cell lines LoVo and Hct116 were obtained from the American Type Culture Collection (Manassas, VA, USA) and cultured at 37 °C in a 5% carbon dioxide (CO_2_) incubator. The cultural conditions were as previously described 16.

### 3. Real-time polymerase chain reaction (RT-PCR) analysis

Detailed information on the real-time polymerase chain reaction (RT-PCR) analysis is provided in the Supplementary Materials and Methods. The sequences of the RT-PCR primers are listed in Supplementary Table 1.

### 4. Western blot assay

*PPFIA1, PPFIA3,* and *PPFIA4* protein expression in NCM460, LoVo, and Hct116 cells and *PPFIA4* protein expression in LoVo and Hct116 cells transfected with and without miRNA mimics and inhibitors were analyzed by western blotting. Cells were collected and lysed in cold lysis buffer (RIPA, Solarbio, China) for 30 minutes. Electrophoresis was performed to separate the samples on 8% sodium dodecyl sulfate-polyacrylamide gels. The separated proteins were transferred to polyvinylidene fluoride or polyvinylidene difluoride (PVDF) membranes (Millipore, USA). At room temperature, 5% defatted milk (BD Biosciences) was used to block the membranes containing proteins for two hours. The PVDF membranes were then incubated with the primary antibodies (Supplementary Table 2) at 4 °C for 16 hours. The next day, secondary antibodies reacted with the primary antibodies for 1.5 hours at room temperature. A ChemiDoc imaging system (Bio-Rad) was used to assess protein expression.

### 5. Relapse and survival analyses

GEPIA (http://gepia.cancer-pku.cn/detail.php) and the TCGA portal (http://www.tcgaportal.org) were used to analyze the correlation between PPFIA mRNA expression and survival time of patients with CRC.

### 6. CRC tissue samples

Paraffin-embedded human CRC samples (n=219) and non-tumor colorectal tissue samples (n=5) were obtained from the Department of Pathology of Tianjin Union Medical Center. The diagnosis of CRC was confirmed by pathological examination. Based on the differentiation of CRCs and metastasis, these samples were divided into five groups: group I, five cases of colorectal epithelial tissue; group II, 51 cases of well-differentiated CRCs; group III, 54 cases of moderately differentiated CRCs; group IV, 51 cases of poorly differentiated CRCs; and group V, 63 cases of lymph node metastatic foci. The clinical parameters of the 224 cases including 219 cases CRC samples and 5 cases of non-tumor colorectal tissue samples were provided in the Supplementary Table 3. The experiments were approved by the hospital review board of the Tianjin Union Medical Center and the confidentiality of patient information was maintained.

### 7. Immunohistochemical (IHC) staining, scoring and quantification

IHC staining, scoring, and quantification were performed as previously described 16. Detailed information is provided in the Supplementary Materials and Methods.

### 8. Functional enrichment analysis of correlated genes

The top 100 genes that were positively correlated with *PPFIA4* in the CRC were selected from the UALCAN database. R software was employed to perform the Gene Ontology (GO) and Kyoto Encyclopedia of Genes and Genomes (KEGG) pathway enrichment analyses.

### 9. Protein-protein interaction (PPI) networks and identification of candidate miRNAs (micro-RNA) and miRNA-mRNA regulation networks

A PPI network of 100 correlated genes was visualized using the STRING database. The Encyclopedia of RNA Interactomes (ENCORI; http://starbase.sysu.edu.cn/) was used to analyze the miRNA-ncRNA, miRNA-mRNA, ncRNA-RNA, RNA-RNA, RBP-ncRNA, and RBP-mRNA interactions from CLIP-seq, degradome-seq, and RNA-RNA interactome data. In our study, ENCORI was used to predict miRNAs that regulate *PPFIA4.* Cytoscape (version 3.7.2) was used to analyze the miRNA-*PPFIA4* network 17.

### 10. Transient transfection

Inhibitors and mimics targeting miR-485-5P were synthesized by Gene-Pharma (Shanghai, China). Detailed information on the sequences is provided in Supplementary Table 4.

## Results

### 1. *PPFIA* mRNA and protein expression levels were associated with the development of CRC

**Figure 1:**
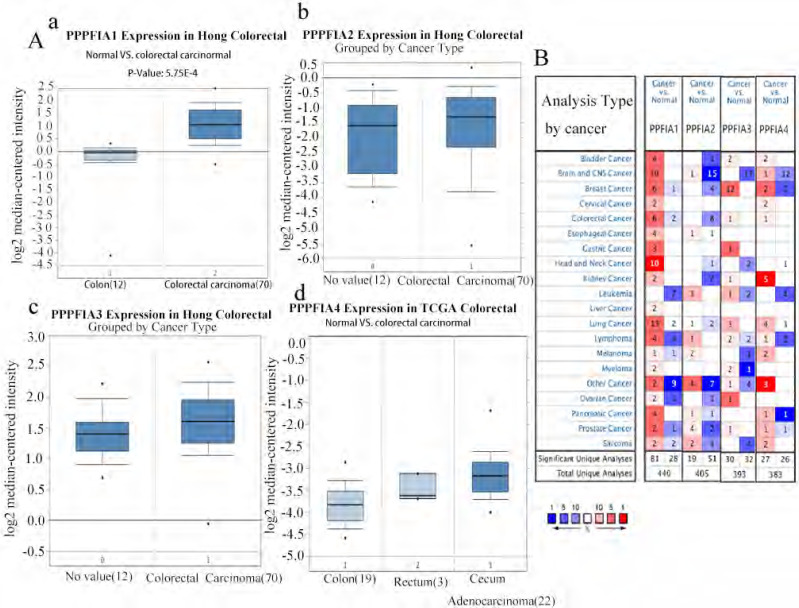
** mRNA expression levels of *PPFIA1, PPFIA2, PPFIA3,* and *PPFIA4* in different types of cancers and CRC. A.** mRNA expression levels of *PPFIA1* (a), *PPFIA2* (b), *PPFIA3* (c) and *PPFIA4* (d) in normal colon and CRC samples as determined by Oncomine databases. **B.** mRNA expression levels of *PPFIA1, PPFIA2, PPFIA3,* and *PPFIA4* in various types of cancers based on Oncomine database analysis. CRC: colorectal cancer.

**Figure 2:**
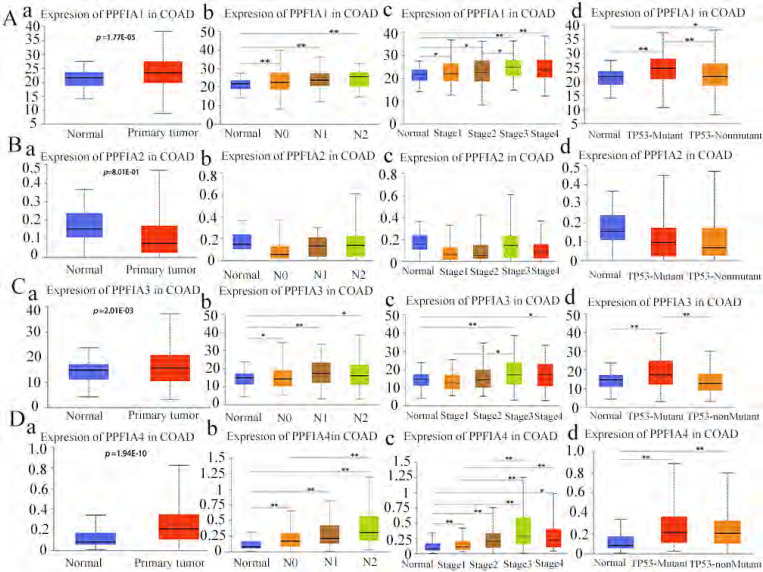
*PPFIA1* (**a**), *PPFIA2* (**b**), *PPFIA3* (**c**) and *PPFIA4* (**d**) mRNA expression levels were associated with lymphatic metastasis, pathological stage, and *TP53*-muation status in CRCs from the UALCAN database (*P < 0.05, ** P < 0.01).

**Figure 3:**
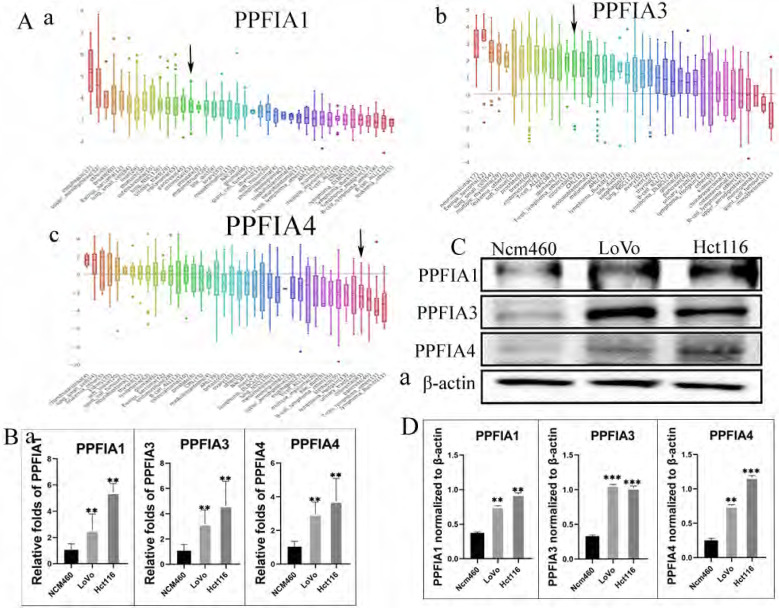
** A.** mRNA expression levels of *PPFIA1* (a), *PPFIA3* (b), and *PPFIA4* (c) in CRC cell lines. Black arrow points to the CRC cell lines. **B.** Statistical chart of the mRNA expression levels of *PPFIA1* (a), *PPFIA3* (b), and *PPFIA4* (c) in LoVo and Hct116 compared with normal colon cell line (NCM460) determined by RT-PCR. **C.** The results of the western blot showed the expression of *PPFIA1, PPFIA3* and *PPFIA4* in LoVo and Hct116, and NCM460. **D.** The histogram revealed that the expression of *PPFIA1* (a), *PPFIA3* (c) and *PPFIA4* (d) is statistically significant between LoVo, Hct116 and NCM460 (**P < 0.01, ***P < 0.001). CRC: colorectal cancer, RT-PCR: reverse transcription polymerase chain reaction.

The Oncomine and UALCAN databases were used to compare the expression of the mRNA level expression of *PPFIA* in human CRC samples and normal colon epithelium samples. The results from the two databases demonstrated that the mRNA expression levels of *PPFIA1, PPFIA3,* and *PPFIA4* were significantly higher in CRC samples than in normal colon epithelium. *PPFIA2* mRNA expression was not significantly different between normal colon epithelial samples and CRC tissues (Figures 1A-a to d, 1B, 2A-a, 2B-a, 2C-a, 2D-a). In addition, the mRNA expression levels of *PPFIA1, PPFIA3*, and *PPFIA4* were associated with tumor stage, lymph node metastasis, and *TP53*-mutation status (Figures 2A-b to d, 2B-b to d, 2C-b to d, and 2D-b to d). Results of CCLE database analysis also confirmed that the mRNA expression levels of *PPFIA1, PPFIA3*, and *PPFIA4* in CRC cell lines were higher than those in normal colon cell lines (Figure 3A - a to c). Subsequently, RT-PCR and western blotting were performed to detect the mRNA and protein levels of *PPFIA1, PPFIA3*, and *PPFIA4* in normal colon (NCM460) and CRC (LoVo and Hct116) cell lines. The results confirmed that both mRNA (Figure 3B - a to c) and protein (Figures 3C and 3D- a to c) expression of *PPFIA1, PPFIA3,* and *PPFIA4* were increased in CRC cell lines compared to the normal colon cell line, and the differences were statistically significant (**P < 0.01, ***P < 0.001).

### 2. Prognostic value of *PPFIA* mRNA expression levels in CRCs

**Figure 4:**
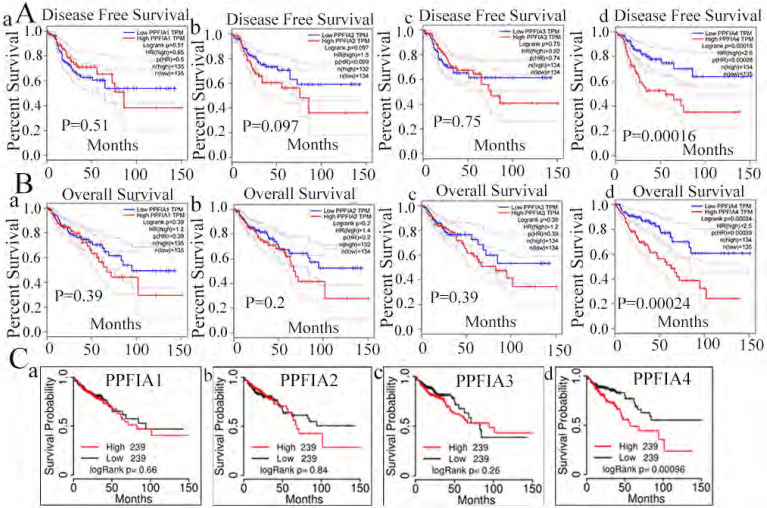
*PPFIA1, PPFIA2, PPFIA3,* and *PPFIA4* prognostic value in CRC patients. **A.** Based on GEPIA, disease free survival of *PPFIA1* (a), *PPFIA2* (b), *PPFIA3* (c) and *PPFIA4* (d) in CRC patients. **B.** Overall survival of *PPFIA1* (a), *PPFIA2* (b), *PPFIA3* (c) and *PPFIA4* (d) in CRC patients based on GEPIA. **C.** TCGA portal-based survival curves for *PPFIA1* (a), *PPFIA2* (b), *PPFIA3* (c) and *PPFIA4* (d) in CRC patients. CRC: colorectal cancer, GEPIA: gene expression profiling interactive analysis.

To evaluate the prognostic value *of PPFIA1, PPFIA2, PPFIA3*, and *PPFIA4* in CRC patients, the GEPIA server and TCGAportal database were used to analyze the survival of CRC patients. The results indicated that the mRNA expression levels of *PPFIA1, PPFIA2,* and *PPFIA3* were not significantly correlated with overall survival (OS) or disease-free survival (DFS) in CRC patients (Figures 4A-a to c, 4B-a to c). However, patients with high *PPFIA4* mRNA expression levels of CRCs had shorter DFS and OS than those with low *PPFIA4* mRNA expression levels, and the difference was statistically significant (Figure 4A-d,* P* = 0.00028; Figure 4C, *P* = 0.00039). These results are consistent with the TCGA portal database (Figure 4C). The results from the two databases demonstrated that high *PPFIA4* mRNA expression levels were associated with poor prognosis in patients with CRC.

### 3. *PPFIA4* IHC staining in human CRC tissues

**Figure 5:**
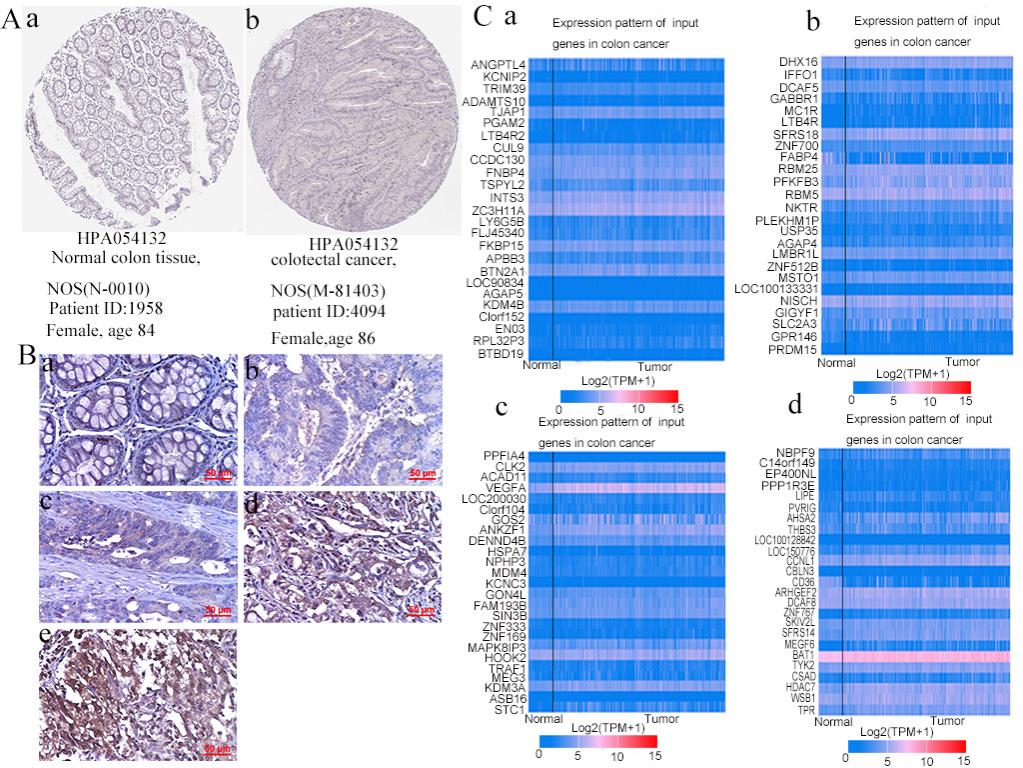
** A.** *PPFIA4* expression in normal colon tissues and CRC tissues was determined using the human protein atlas. **B.** Immunohistochemical staining was performed to detect the expression of *PPFIA4* in human normal colon and CRC tissues (400×). (a) Normal human colon tissues; (b) well-differentiated CRC; (c) moderately differentiated CRC; (d) poorly differentiated CRC; (e) Lymph node foci. **C.** Top 100 genes co-expressed with *PPFIA4* in CRC based on UALCAN. a. Top 25 genes were co-expressed with *PPFIA4* in CRC based on UALCAN. b. From 26 to 50 genes co-expressed with *PPFIA4* in CRC based on UALCAN. c. From 51 to 75 genes were co-expressed with *PPFIA4* in CRC based on UALCAN. d. From 76 to 100 genes were co-expressed with *PPFIA4* in CRC based on UALCAN. CRC: colorectal cancer.

*PPFIA4* was selected for further study based on the results of the bioinformatics analysis described above. According to the Human Protein Atlas website, IHC analysis showed that the expression of *PPFIA4* protein was higher in CRC tissues than in normal tissues (Figure 5A). In this study, IHC analysis was performed to detect the protein expression of *PPFIA4* in five normal colon tissue samples and 219 cases. The results showed that the protein expression of *PPFIA4* was higher in CRC tissues than in normal colon tissues (Figures 5B-a to e and Table 1). The expression of *PPFIA4* was significantly lower in Group I than in Groups II (*P* = 0.009), III (*P* = 0.029), IV (*P* = 0.000), and V (*P* = 0.000). *PPFIA4* expression was higher in groups IV (*P* = 0.000) and V (*P* = 0.000) than in group III. In addition, *PPFIA4* expression was also higher in groups V (*P* = 0.004) and IV (*P* = 0.000) than in group II. The differences were not statistically significant between groups II and III (*P* = 0.299) or between groups IV and V (*P* = 0.128).

### 4. *PPFIA4* gene co-expression and functional enrichment analysis

**Figure 6:**
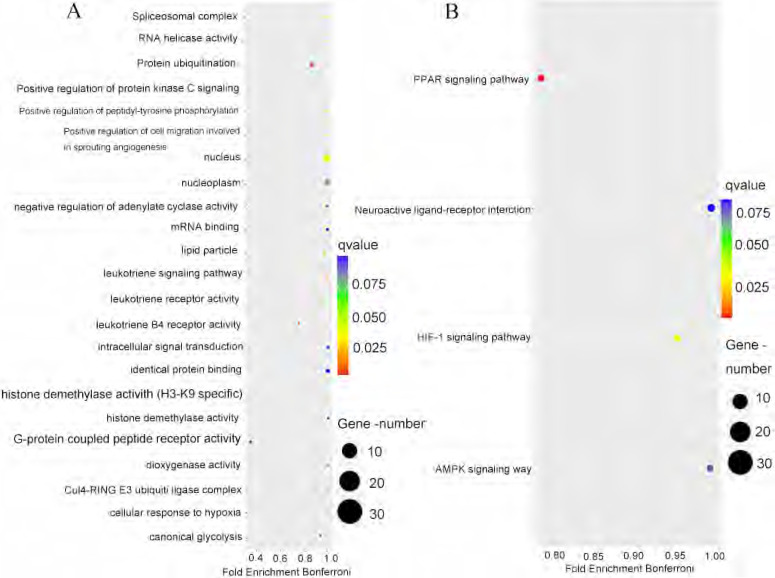
** The enrichment analysis of the top 100 genes co-expressed with *PPFIA4.* A.** GO analysis of the top 100 genes co-expressed with *PPFIA4*.** B.** KEGG analysis of the top 100 genes co-expressed with *PPFIA4*. KEGG: Kyoto Encyclopedia of Genes and Genomes, GO: Gene Ontology.

To better understand the biological processes and signaling pathways that are involved in *PPFIA4*, co-expressed genes with *PPFIA4* were analyzed. The UALCAN database was used to identify the top 100 genes that were positively correlated with *PPFIA4* expression in CRC (Figures 5C-a to d). Consequently, the PPI network was generated in the STRING protein interaction database to show the interaction among the 100 genes (Supplementary Figure 1), and then the 100 genes were imported into the Cytoscape platform (Version 3.7.1) to clarify the mutual relations of the 100 top genes co-expressed with *PPFIA4* in CRC (Supplementary Figure 2). Gene Ontology (GO) and Kyoto Encyclopedia of Genes and Genomes (KEGG) pathway enrichment analyses showed that the co-expressed genes of *PPFIA4* were significantly enriched in G-protein coupled peptide receptor activity and leukotrience B4 receptor activity (Figure 6A). The most enriched KEGG pathways were the PPAR and HIF-1a signaling pathways (Figure 6B).

### 5. miRNAs and the expression of *PPFIA4*

**Figure 7:**
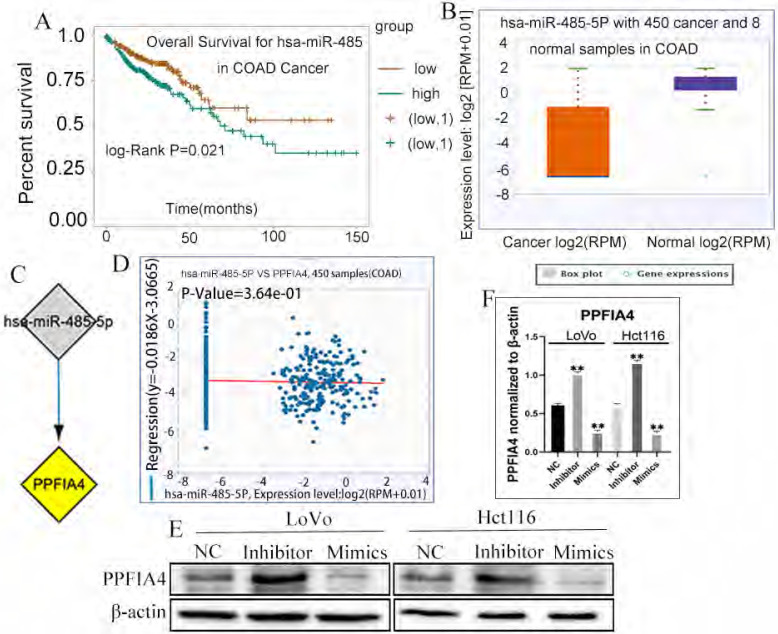
** A.** Overall survival of miR-485-5p in CRC patients based on the ENCORI database. **B.** ENCORI determined the expression of miR-485-5p in normal colon and CRC samples. **C.** miRNA-*PPFIA4* regulation network (Cytoscape). **D.** The negative correlation between miR-485-5p and *PPFIA4* expression was determined by ENCORI. **E.** The expression of *PPFIA4* in LoVo and Hct116 after treatment with inhibitors and mimics targeting miR-485-5p. **F.** The statistical chat of *PPFIA4* expression in LoVo and Hct116 after treatment with inhibitors and mimics targeting miR-485-5p (**P < 0.01). CRC: colorectal cancer, ENCORI, encyclopedia of RNA Interactomes.

miRNAs are a class of small non-coding RNAs that play a critical role in the initiation and progression of diverse tumors 18, 19. To determine the correlation between miRNAs and *PPFIA4*, the ENCORI platform was used to predict the miRNAs involved in the regulation of *PPFIA4*. Eventually, it was demonstrated that a total of 17 miRNAs could regulate the expression of *PPFIA4* (Table 2). Among these, 14 miRNAs were negatively correlated with *PPFIA4* expression. Four miRNAs among the 14 miRNAs showed decreased expression in CRCs, and only miR-485-5P was significantly associated with the prognosis of CRC patients (Figure 7A, *P* = 0.021). The results showed that miR-485-5P expression was decreased in CRC samples compared to that in normal colon tissue samples and negatively regulated the expression of *PPFIA4*, as determined by the ENCORI database (Figures 7B to 7D).

To further verify whether miR-485-5P can regulate the expression of *PPFIA4*, the expression of *PPFIA4* was detected in LoVo and Hct116 cells after transfection with miR-485-5P inhibitor and miR-485-5P mimics. Intriguingly, the results indicated that *PPFIA4* expression was decreased in LoVo and Hct116 cells after treatment with miR-485-5P mimics and increased after treatment with miR-485-5P inhibitors. These differences were statistically significant (Figure 7E and 7F, **P < 0.01). The results showed that miR-485-5P negatively modulated the expression of *PPFIA4*.

## Discussion

High expression of *PPFIA1* has been demonstrated in several cancers, including breast cancer, head and neck squamous cell carcinomas, and oropharyngeal carcinomas 20-28. In studies, liprin-α1 has been shown to promote tumor cell migration and invasion 29, 30. Inhibition of liprin-α1 resulted in increased expression of the transmembrane protein CD82, which plays an essential role in suppressing metastasis in several solid tumors 31. Liprin-α2, encoded by *PPFIA2*, is located in the mature hippocampal presynapses. Liprin-α2 plays an important role in regulating neuronal activity and is highly expressed in the urinary sediments of patients with prostate cancer 9, 32-34. *PPFIA3* can be methylated in gastric cancer but is rarely methylated in normal gastric tissues. Furthermore, it has an independent prognostic value in patients with pancreatic cancer 10-12. A study indicated that *PPFIA4* was highly expressed in clear renal cell cancer compared to that in normal kidney tissue 35. Xu *et al.* 38 showed that *PPFIA4* was upregulated in human thyroid cancer tissues compared to nodular goitre tissues 36. Additionally, *PPFIA4* expression was significantly increased in castration-resistant prostate cancer tissues compared to that in prostate cancer 37. Wang *et al.* 40 showed that *PPFIA4* might be a potential biomarker for the diagnosis of pilocytic astrocytoma 38. Results of Huang J, *et al* confirmed that PPFIA4 upregulation correlated with poor prognosis and higher clinical stages of CRC patients. Overexpression of PPFIA4 could increase the expression of EMT-related proteins and promote the proliferation, migration and invasion of colorectal cancer cells 39. Bioinformatics analysis from Oncomine and UALCAN databases revealed that mRNA expression levels of *PPFIA1, PPFIA3*, and *PPFIA4* were higher in CRC samples than in normal colon tissue. Evidence from CCLE databases and RT-PCR results also indicated that the mRNA of *PPFIA1, PPFIA3,* and *PPFIA4* was overexpressed in the CRC cell lines LoVo and Hct116. The mRNA levels of *PPFIA1, PPFIA3*, and *PPFIA4* were associated with tumor stage, lymph node metastasis, and *TP53*-mutation status. More importantly, survival analysis determined by TCGA portal and GEPIA illustrated that *PPFIA4* expression was associated with the prognosis of patients with CRC. IHC staining analysis showed that *PPFIA4* was closely associated with the degree of malignancy in CRCs.

To identify the biological processes and signal pathways of *PPFIA4* involving in CRCs, the top 100 genes co-expressed with *PPFIA4* were entered into the STRING database and Cytoscape software to obtain the PPI network. A gene set enrichment analysis was performed to study the role of genes co-expressed with *PPFIA4* in CRCs. The GO enrichment analysis results indicated that these genes mainly participated in G-protein coupled peptide receptor activity and leukotriene B4 receptor activity. Furthermore, KEGG pathway enrichment analysis revealed that the co-expressed genes were enriched in PPAR and HIF-1 signaling pathways. Under hypoxia, *PPFIA4* can promote the proliferation of cancer cells *via* mitogen-activated protein kinase (MAPK) or phosphoinositide 3-kinase (PI3K) signaling pathways. It enhances invasion ability through the epithelial-mesenchymal transition in pancreatic cancer. Moreover, *PPFIA4* can promote chemotherapy resistance through MAPK pathways *via* HIF-1α expression in small cell lung cancer 14, 15. Mattauch *et al*. revealed that *PPFIA4* could be directly modulated by HIF-1α and by stabilizing E-cadherin and β-catenin to regulate cell junctions in renal cell carcinoma and breast cancer 35. *PPFIA4* also protects against nickel-induced cytotoxicity and modulates receptor protein tyrosine phosphatase-leukocyte antigen related receptor F (RPTP-LAR) activity 40. *PPFIA4* promotes castration-resistant prostate cancer progression *via* methylenetetrahydrofolate dehydrogenase 2 through mitochondrial metabolism 37.

miRNAs are single-stranded, evolutionarily conserved molecules that modulate a wide range of target genes at the post-transcriptional level in physiological and pathological processes in humans 41, 42. A recent study showed that miR-485-5p expression was decreased in CRC cell lines in comparison with the normal colon epithelial cell line, and in CRC tissues compared to paired para-cancerous tissues. MiR-485-5p overexpression suppressed the proliferation, migration, and invasion of CRC cells. CD147 was negatively regulated by miR-485-5p through binding a conserved sequence specifically within the CD147 43. miR-485-5p, which serves as a tumor suppressor, has been shown to be a potential prognostic biomarker for CRCs 19. The expression of miR-485-5p could be sponged by LINC01224 and LINC01224 knockdown could increase the apoptosis of CRC cells 44. In CRC, miR-485-5p plays a key role in targeting multiple glioma genes. In addition, HIF-1α can target the miR-485-5p promoter region to inhibit transcription 45. Bioinformatic analysis showed that the expression of miR-485-5P was closely associated with the prognosis of CRC patients. The expression of *PPFIA4* increased in LoVo and Hct116 cells when treated with miR-485-5P mimics and decreased when treated with miR-485-5P inhibitors. These results proved that miR-485-5P negatively regulates the expression of *PPFIA4*.

Our research indicated that *PPFIA1, PPFIA3,* and *PPFIA4* expression increased in the CRC cell lines LoVo and Hct116. *PPFIA4,* which is involved in the PPAR and HIF-1 signaling pathways, is associated with the degree of malignancy of CRCs and could be used to evaluate the prognosis of patients with CRC. Thus, *PPFIA4* may be a candidate biomarker and therapeutic target for CRCs. More studies on the potential mechanisms by which *PPFIA4* regulates the development of CRC are needed in the future.

## Supplementary Material

Supplementary material and methods, figures, and tables.

## Figures and Tables

**Table 1:** Comparison of the *PPFIA4* staining index among different groups of human CRCs

	Group	n	Staining index	Value of statistics	P*
Normal colon tissue	I	5	2.2±0.83666	χ^2^ = 53.837	0.000
Well-differentiated CRCs	II	51	5.5882±3.11882		
Moderately differentiated CRCs	III	54	4.8519±2.17540		
Poorly differentiated CRCs	IV	51	7.6471±3.56552		
Metastatic lymph node foci	V	63	8.6349 ±3.27897		

*P<0.05: statistically significant. (*P*: difference between the three groups;* P*1 (difference between groups I and II) = 0.009; *P*2 (difference between groups I and III) = 0.029; *P*3(difference between groups I and IV) = 0.000; *P*4(difference between groups I and V) = 0.000. *P*5 (difference between groups II and III) = 0.299.* P*6 (difference between Groups III and IV) = 0.000.* P*7 (difference between Groups III and V) = 0.000.* P*8 (difference between Groups II and IV) = 0.004.* P*9 (difference between groups II and V) = 0.000.* P*10 (difference between Groups IV and V) = 0.128. CRC, colorectal cancer.

**Table 2:** Micro-RNAs targeted *PPFIA4* in ENCORI

Number	miRNA	Coefficient-R	p-value
1	hsa-miR-101-3p	-0.051	2.76E-01
2	hsa-miR-144-3p	-0.087	6.41E-02
3	hsa-miR-329-3p	-0.019	6.86E-01
4	hsa-miR-485-5p	-0.043	3.64E-01
5	hsa-miR-580-3p	-0.022	6.42E-01
6	hsa-miR-6884-5p	-0.097	4.06E-02
7	hsa-miR-650	-0.046	3.28E-01
8	hsa-miR-660-5p	-0.032	4.94E-01
9	hsa-miR-502-3p	-0.001	9.85E-01
10	hsa-miR-362-3p	-0.131	5.41E-03
11	hsa-miR-501-3p	-0.004	9.25E-01
12	hsa-miR-138-5p	0.021	6.59E-01
13	hsa-miR-668-3p	0.058	2.19E-01
14	hsa-miR-134-5p	0.076	1.06E-01
15	hsa-miR-342-3p	0.114	1.52E-02
16	hsa-miR-423-3p	0.09	5.54E-02
17	hsa-miR-582-5p	0.012	7.92E-01

## Abbreviations

CRC: colorectal cancer
GO: gene ontology
KEGG: kyoto encyclopedia of genes and genomes
CCLE: cancer cell line encyclopedia
GEPIA: gene expression profiling interactive analysis
ENCCRCORI: encyclopedia of RNA interactomes
OS: overall survival
DFS: disease free survival
IHC: immunohistochemical
miRNAs: micro-RNA
MAPK: mitogen-activated protein kinases
PI3K: phosphoinositide 3-kinase
RT-PCR: reverse transcription polymerase chain reaction
PPI: Protein-protein interaction
LAR: leukocyte common antigen-related gene
