# Supplementary material for: Clinicopathological Significances and Prognostic Value of PPFIA4 in Colorectal Cancer
Source: J Cancer. 2023 Jan 1;14(1):24–34. doi: 10.7150/jca.78634 (PMC9809326; doi:10.7150/jca.78634)
Supplement: Supplementary file 1 — Supplementary material and methods, figures, and tables. [file jcav14p0024s1.pdf]

Supplementary table 1. Primers used in this study.

Names	Sense (5′-3′)	Anti-sense(5′-3′)
PPFIA1	ATGATGTGCGAGGTGATGCC	GGCGGTCCCTTTCTTCTAGC
PPFIA3	ACAGGACGGGTTGGCTACA	AAGTTCAGCTCCTTCGTCAGA
PPFIA4	GAAACACCAGCTGCTTGAA	TGATGGCACCACTCTTGAC
GAPDH	ACCACAGTCCATGCCATCAC	TCCACCACCCTGTTGCTGTA

Supplementary table 2. Detailed information of the antibodies used in this study.

**Antibody**	**Company**	**Dilution**
PPFIA1	Bioss	WB,1:500
PPFIA3	ABclonal	WB,1:500
PPFIA4	biorbyt	WB,1:200;IHC,1:100
β-actin	Zhongshan	1:1000(WB)
Anti-rabbit IgG	Cell Signaling Technology	1:3000(WB)
Anti-mouse IgG	Cell Signaling Technology	1:3000(WB)

WB: Western blot； IHC: Immunohistochemical staining

Supplementary table 3. The clinical parameters of 219 cases CRC samples (n=219) and non-tumor colorectal tissue samples (n=5).

		Colorectal cancer (n=219)	Normal colon(n=5)
Gender	male	119	1
	female	100	4
Age	≤60	107	3
	＞60	112	2
T	T1+T2	33	
	T3+T4	186	
Lymph node metastasis	yes	111	
	no	108	
Dukes' stage	A+B	107	
	C	112	

Supplementary table 4. The sequences of miR-485-5p mimic and inhibitor.

Names	Sense (5′-3′)	Anti-sense(5′-3′)
miR-485-5p-mimic	AGAGGCUGGCCGUGAUGAA UUC	AUUCAUCACGGCCAGCCUC UUU
miR-485-5p-inhibi	GAAUUCAUCACGGCCAGCCU	

tor	CU
microRNA inhibitor N.C	CAGUACUUUUGUGUAGUAC AA

Supplementary Materials and Methods

Real-time PCR analysis

Total RNA from NCM460, LoVo and Hct116 cell were isolated using TRizol reagent (Invitrogen, USA) and reversely transcripted into cDNA according to the manufacturer's instructions (Novcare Biotech, China). For PCR, each reaction contained 10 μL of 2× ChamQ SYBR (Vazyme, Q331-AA), 2 μL of cDNA diluted in nuclease free water, 1ul of 10 μM primer with nuclease-free water were added to a total volume of 20 μL. Amplification and detection were performed using the ABI Prism 7500 sequencer. Glyceraldehyde 3-phosphate dehydrogenase (GAPDH) was the reference genes.

Immunohistochemical (IHC) staining, scoring, and quantification

Xylene was used to dewax the paraffin-embedded CRC tissue, which was then rehydrated with gradient alcohol solutions. To restore antigens, sections were immersed in boiled citrate buffer solution (Zhongshan Inc.) for 3 minutes. Next, endogenous peroxidase activity was blocked by endogenous peroxidase inhibitor followed with goat serum blocking the nonspecific background. Then these sections were incubated with primary antibody against PPFIA4 at 4°C overnight. And next day incubated with a secondary antibody for 1 h. Horseradish peroxidase-labeled streptomycin (Zhongshan Inc.) was then added to cells for 30 min. The antigen was quantified according to two parameters: one is staining strength ranging from light yellow to dark brown; the other is 79the number of positive stained cells. Thereby, PPFIA4 positivity was graded based on the number and strength 3of positive cells: The staining intensity was assessed as follows: 0, negative (no staining); 1+, weakly positive (faint yellow staining); 2+, moderately positive (brownish-yellow staining); and 3+, strongly positive (brown staining). The number of positive cells was visually evaluated as follows: 0=0-5% (negative); 1+=6%-25% (weak); 2+=26%-50% (moderate); 3+=51%-75% (above moderate); and 4+=76%-100% (strong).

Supplementary figure and figure legend

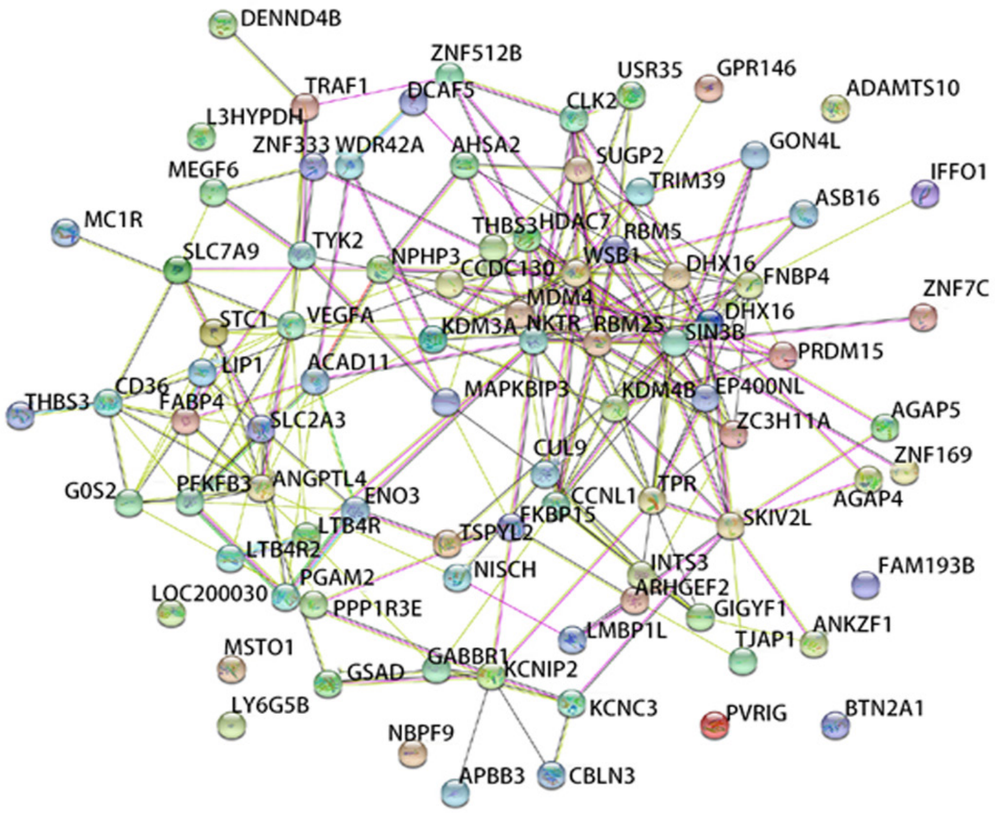

Supplementary figure 1: Mutual relations of the 100 top genes co-expressed with *PPFIA4* in CRC using a string database.

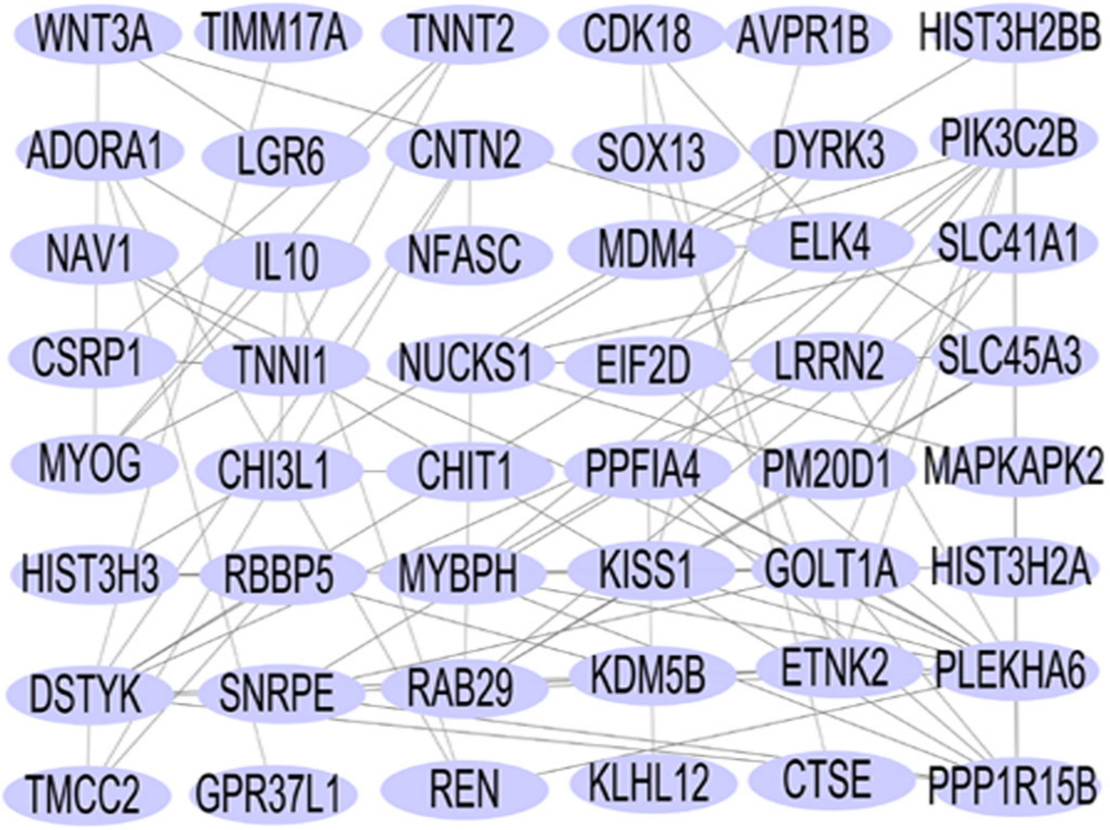

Supplementary figure 2: The mutual relations of the 100 top genes co-expressed with *PPFIA4* in CRC were visualized on the Cytoscape platform.
